# Supplementary material for: Publication bias in pharmacogenetics of adverse reaction to antiseizure drugs: An umbrella review and a meta-epidemiological study
Source: PLoS One. 2022 Dec 30;17(12):e0278839. doi: 10.1371/journal.pone.0278839 (PMC9803138; doi:10.1371/journal.pone.0278839)
Supplement: S1 File — (DOCX) [file pone.0278839.s001.docx]

SUPPLEMENTARY MATERIALS OF

Publication bias in pharmacogenetics of adverse reaction to antiseizuredrugs: an umbrella review and a meta-epidemiological study

1. Equator’s Guidelines for reporting meta-epidemiological methodology research’s checklist.
2. Search strategy.
3. Figure S1. Funnel plots generated for each association _[genotype-drug-ADR]_ combining the published meta-analyses.
4. Figure S2. Funnel plots generated combining the association A, B and C ([HLA-B*15:02 – carbamazepine – SCR/SJS/SJS,TEN] (potential duplicates not removed).
5. Results of individual studies

**Items for reporting** methodology research proposed by the Equator’s Guidelines for reporting meta-epidemiological methodology research (Murad et al, Guidelines for reporting meta-epidemiological methodology research. Evid Based Med. 2017 Aug;22(4):139–42)

| **Section/topic** | **#** | **Checklist item** | **Reported on page #** |
| --- | --- | --- | --- |
| **TITLE** | | |  |
| Title | 1 | Identify the report as a meta-epidemiologic study | 1 |
| **ABSTRACT** | | |  |
| Structured summary | 2 | Provide a structured summary that includes the background of the topic, goal of the study, data sources, method of data selection, appraisal and synthesis methods, results, limitations, conclusions and implications of key findings | 3 |
| **INTRODUCTION** | | |  |
| Rationale | 3 | Describe the rationale for the meta-epidemiological study in the context of what is already  known | 4 |
| Objectives | 4 | Provide an explicit statement of the goal of the meta-epidemiological study and the hypothesis being empirically tested | 4-5 |
| **METHODS** | | |  |
| Protocol | 5 | Indicate if a protocol exists, if and where it can be accessed (eg, Web address). Registration of a protocol is not mandatory | 6 |
| Eligibility criteria | 6 | Specify study characteristics used as criteria for eligibility with a rationale | 6 |
| Information sources | 7 | Describe all information sources (eg, databases with dates of coverage, contact with experts to identify additional studies, Internet searches) and search date | 6-7 |
| Search | 8 | Present full electronic search strategy for at least one database, including any limits used, such that it could be repeated. Search is commonly not driven by a clinical question | Supplementary Information.2 |
| Study selection | 9 | Describe the process for selecting studies for inclusion (ie, how many reviewers selected studies, reviewing in duplicate or by single individuals) | 7 |
| Data collection process | 10 | Describe method of data extraction from reports (eg, piloted forms, independently, in duplicate) and any processes used for manipulating data or obtaining and confirming data from investigators | 7 |
| Data items | 11 | List and define all variables for which data were sought and any assumptions and imputations made | 7 |
| Risk of bias in individual studies | 12 | If risk of bias assessment of individual studies was relevant to the analysis, describe the items used and how this information is to be used during data synthesis | 7 |
| Summary measures | 13 | State the principal summary measures (eg, ratio of risk ratios, difference in means) and explain its meaning and direction to readers | 7 |
| Synthesis of results | 14 | Describe the statistical or descriptive methods of synthesis including measures of consistency if relevant. If applicable, describe the development of statistical or simulation modelling based on theoretical background. Describe and justify assumptions and computational approximations. Describe methods of additional analyses (eg, sensitivity or subgroup analyses, meta-regression), if done, indicating which were prespecified | 8 |
| **RESULTS** | | |  |
| Study selection | 17 | Give numbers of studies assessed for eligibility and included in the study, with reasons for exclusions at each stage, ideally with a flow diagram. Present a measure of inter-reviewer agreement (eg, kappa statistic) | Fig 1 |
| Study characteristics | 18 | For each study, present characteristics for which data were extracted and provide the citations. Clinical characteristics may not always be relevant | Table 1 |
| Risk of bias within studies | 19 | If risk of bias assessment of individual studies was used in the meta-epidemiological analysis, report risk of bias indicators of each study to allow replication of findings | 10 |
| Results of individual studies | 20 | Present data elements used in the meta-epidemiological analysis from each study (results of clinical outcomes may not be relevant) | Supplementary Information.4 |
| Synthesis of results | 21 | Present results of statistical analysis done, including measures of precision and measures of consistency. Present validity of assumptions and fit of statistical or simulation modelling, if applicable | 13 |
| Additional analysis | 23 | Give results of additional analyses, if done (eg, sensitivity or subgroup analyses, metaregression) | 16 |
| **DISCUSSION** | | |  |
| Summary of evidence | 24 | Summarise the main findings and compare them with existing knowledge about the topic. The quality of evidence may not be relevant; however, investigators should describe their certainty in the results to readers | 18 |
| Limitations | 25 | Discuss limitations at research methodology level (eg, likelihood of reporting or publication bias) | 19-20 |
| Conclusions | 26 | Provide general interpretation of the results and implications for future research. Provide any plausible impact on clinical practice | 21 |
| **FUNDING** | | |  |
| Funding | 27 | Describe sources of funding for the methodology research and role of funders | 2 |

* Results of the individual studies were used as a data sources to generate our dataset, as each unique association of a triplet _[genotype-drug-ADR]_ may be studied across different published meta-analyses, but not as our outcome of interest *per se*.

**Search strategy**

Pharmacogen* OR Association OR Polymorphism OR Polymorphisms OR Genetic variant OR Genetic variants OR Genetic variation OR Genetic variations OR Genetic association OR Genetic associations OR Allele OR Alleles OR DNA polymorphism OR DNA polymorphisms OR Deoxyribonucleic acid polymorphism OR Deoxyribonucleic acid polymorphisms OR Gene polymorphism OR Gene polymorphisms OR Genetic polymorphism OR Genetic polymorphisms OR Single nucleotide polymorphism OR Single nucleotide polymorphisms OR Candidate gene association OR Candidate gene associations OR Candidate genes association OR candidate genes associations OR Polymorphism single nucleotide OR Genome-wide association studies OR Genome wide association scan OR GWA studies OR GWAS OR Whole genome association analysis OR Whole genome association study OR Genome wide association studies or Genome wide association study OR Genome-wide association study OR Genetic association study OR Genetic association studies

AND

Epilepsy OR Epilepsy treatment OR Epilepsy therapy OR Antiepileptics OR Antiepileptic OR Antiepilepsy drug OR Antiepilepsy drugs OR Antiepileptic drug OR Antiepileptic drugs OR Anticonvulsants OR Anticonvulsant drug OR Anticonvulsant drugs OR Anticonvulsant agent OR Anticonvulsant agents OR Anticonvulsive drug OR Anticonvulsive drugs OR Anticonvulsive agent OR Anticonvulsive agents OR AED OR AEDs OR Anti-seizure OR Anticonvulsant medication OR Anticonvulsant medications OR Anticonvulsant medicine OR Anticonvulsant medicines OR Anticonvulsive medication OR Anticonvulsive medications OR Anticonvulsive medicine OR Anticonvulsive medicines OR Antiepileptic medication OR Antiepeptic medications OR Antiepileptic medicine OR Antiepileptic medicines OR Antiepilepsy medication OR Antiepilepsy medications OR Antiepilepsy medicine OR Antiepilepsy medicines OR Epilepsy drug OR Epilepsy drugs OR Epilepsy agent OR OR Epilepsy agents OR Epilepsy medication OR Epilepsy medications OR Epilepsy medicine OR Epilepsy medicines

AND

Toxicity OR Adverse event OR Adverse events OR Adverse reaction OR Adverse reactions OR Adverse effect OR Adverse effects OR Serious event OR Serious events OR Serious reaction OR Serious reactions OR AE OR AEs OR SAE OR SAEs OR Undesirable effect OR Undesirable effects OR Serious effect OR Serious effects OR Safety OR Negative effect OR Negative effects OR Bad effect OR Bad effects OR Deleterious effect OR Deleterious effects OR Detrimental effect OR Detrimental effects OR injurious effect OR Injurious effects OR harmful effect OR harmful effects OR negative impact OR negative impacts OR Unfavorable effect OR Unfavorable effects OR Unfortunate effect OR Unfortunate effects OR Negative outcomes OR Undesirable reaction OR Undesirable reactions OR Negative reaction OR Negative reactions OR Bad reaction OR Bad reactions OR Deleterious reaction OR deleterious reactions OR Detrimental reaction OR Detrimental reactions OR Injurious reaction OR Injurious reactions OR Harmful reaction OR Harmful reactions OR OR Unfavorable reaction OR Unfavorable reactions OR Unfortunate reaction OR Unfortunate reactions OR Risks OR Threats

AND

Meta-analysis OR Meta-analyses OR Meta analysis OR Meta analyses OR Systematic review

Figure S1. Funnel plots generated for each association _[genotype-drug-ADR]_ combining the published meta-analyses (the association #A is reported in the funnel plot (1), the association #B in the funnel plot (2), and so on).


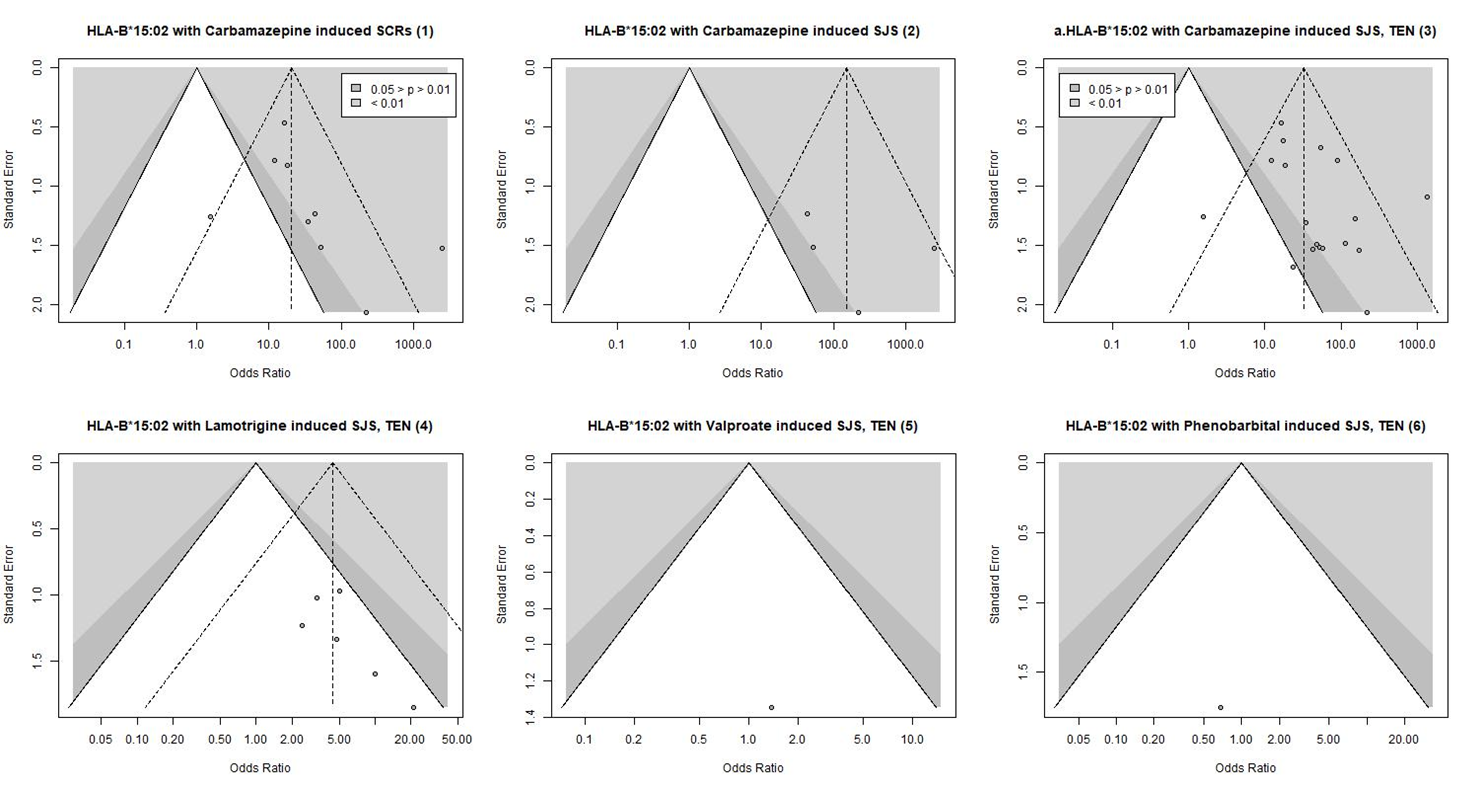


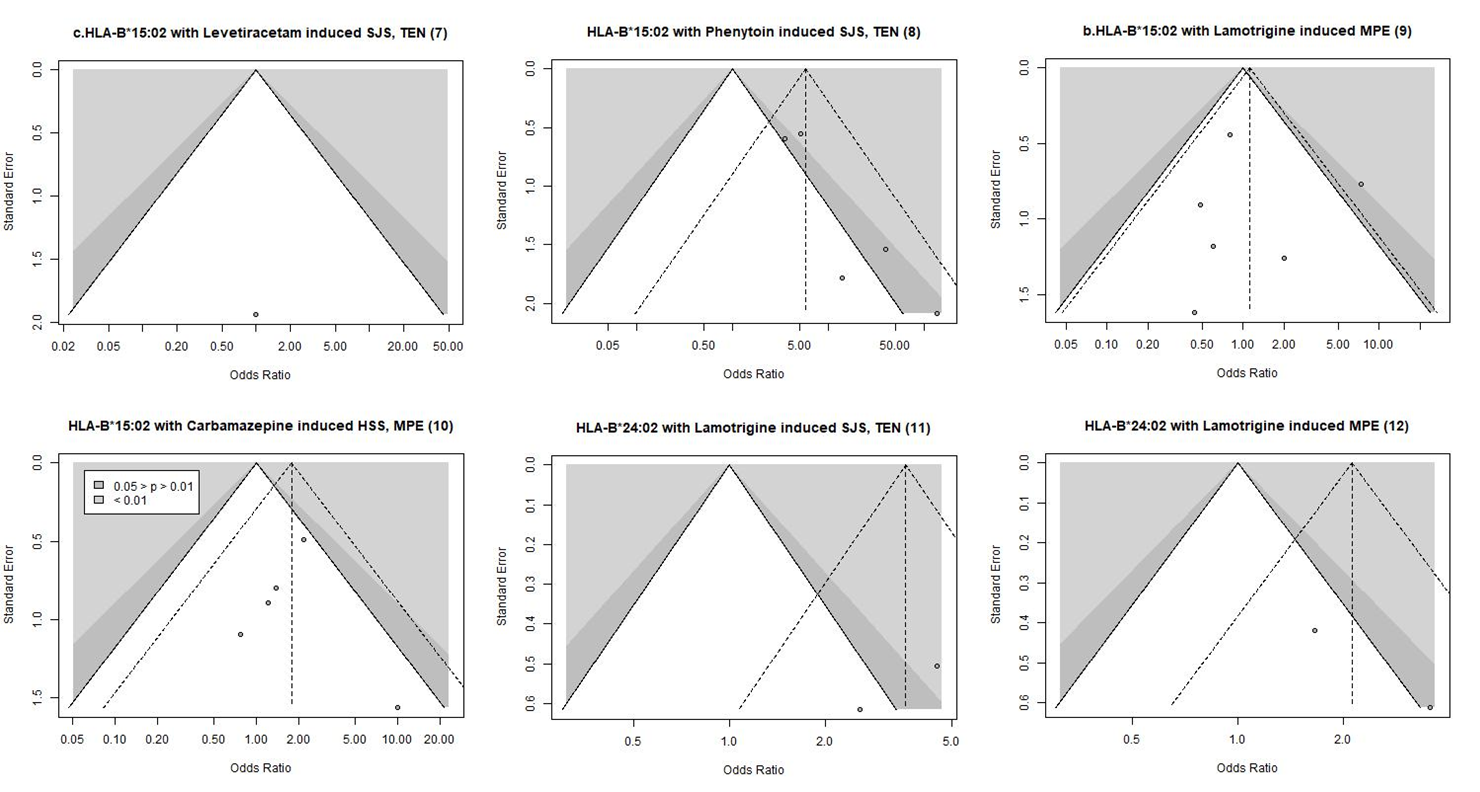


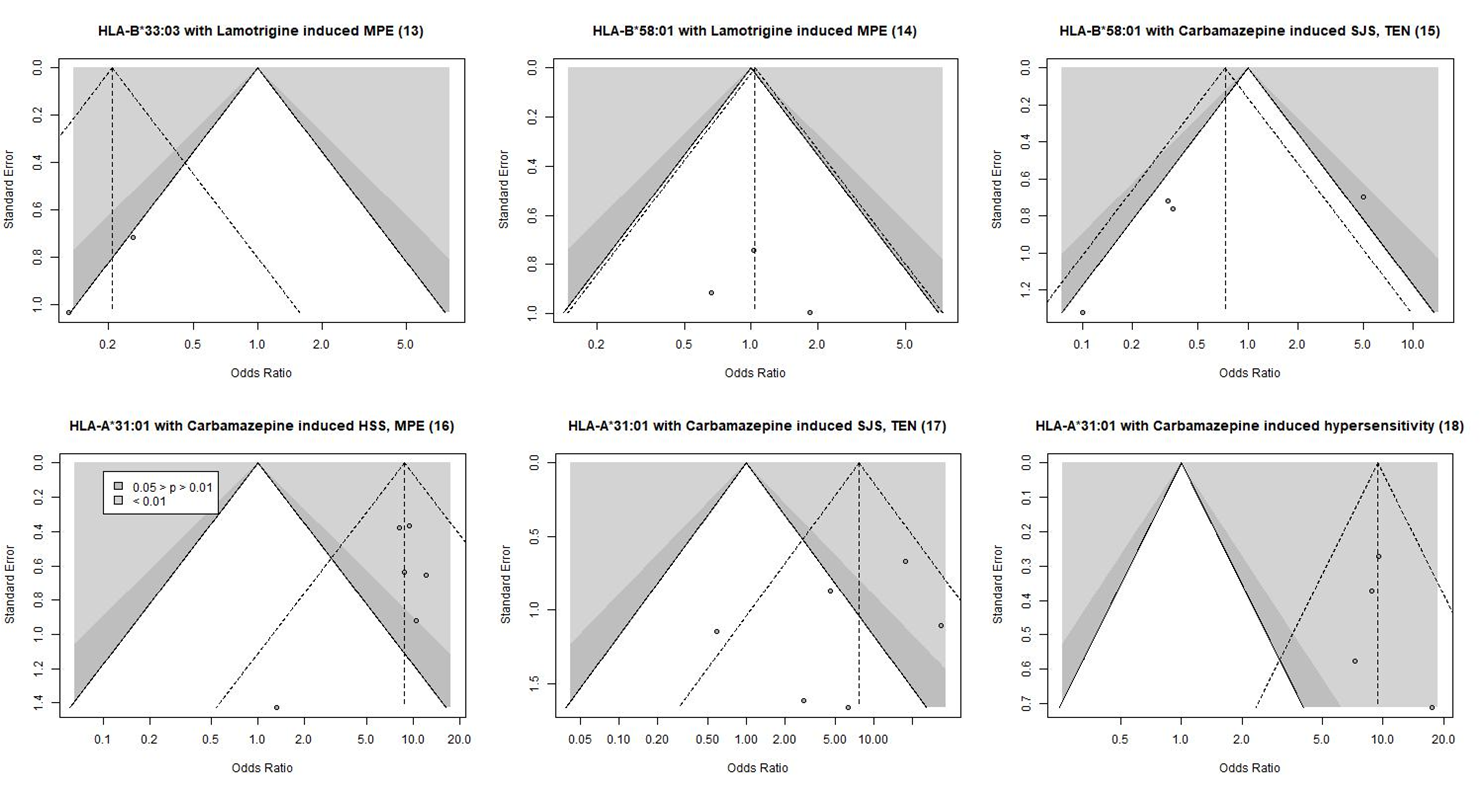


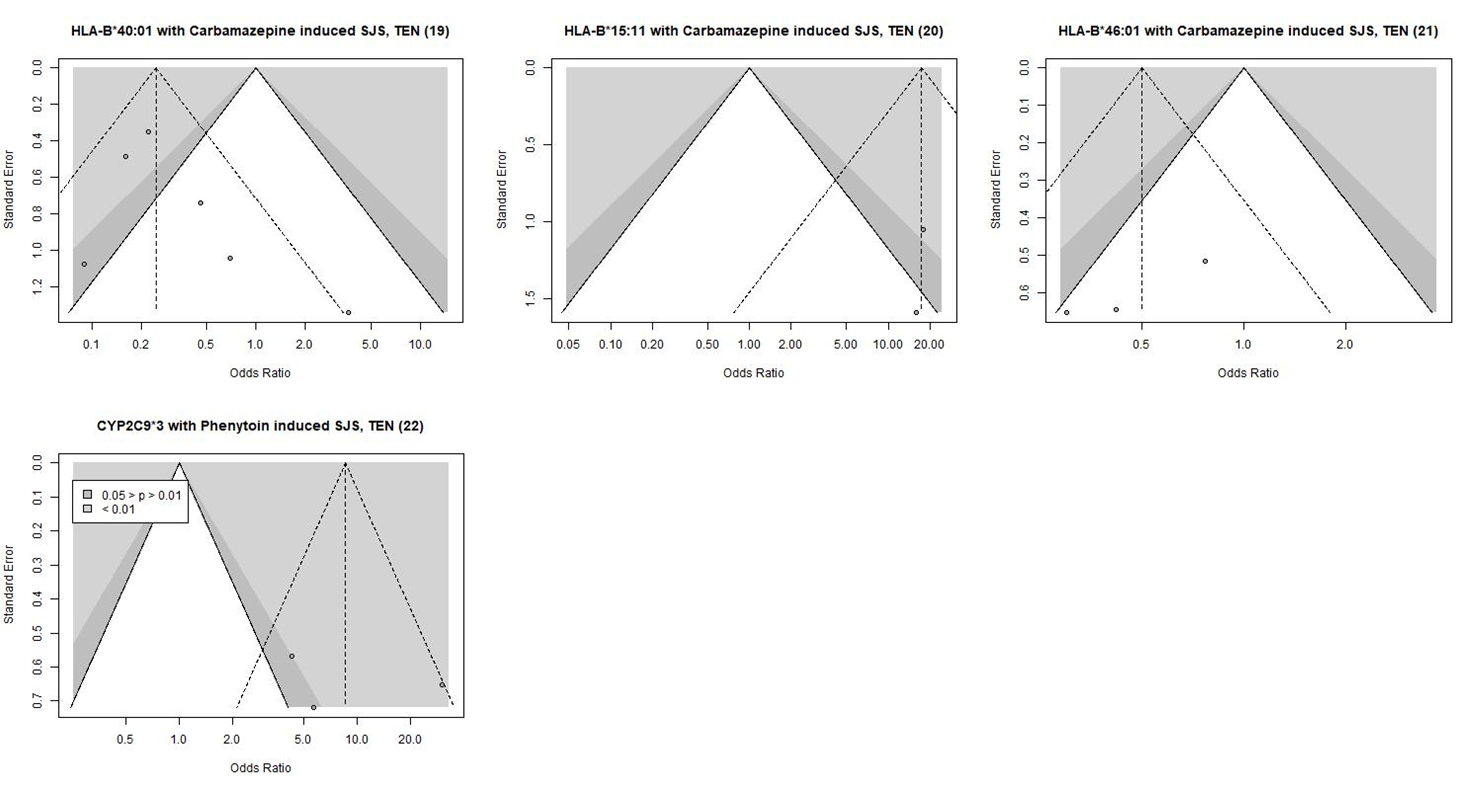


Figure S2. Funnel plots generated combining the association A, B and C ([HLA-B*15:02 – carbamazepine – SCR/SJS/SJS,TEN] (potential duplicates not removed).


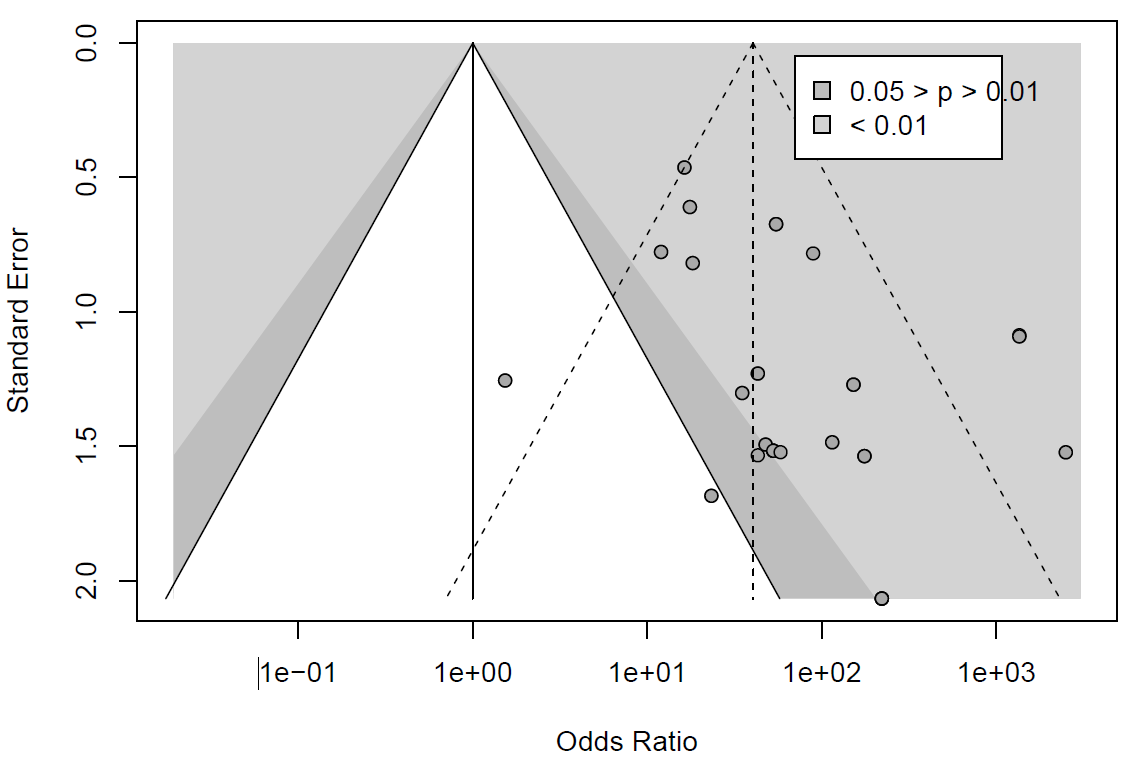


Supplementary Information 4: Results of individual studies (here, published systematic review).

On the right: author’s column of the included systematic review; identification number (IdP) of the 33 published meta-analysis. Rows are ordered by IdP. ADR: adverse drug reaction; HSS: hypersensitive syndrome; MPE: Maculopapular exanthema, SCRs: Serious Cutaneous Reactions, SJS: Stevens - Johnson syndrome, TEN: Toxic Epidermal Necrolysis. Control group: treated tolerant patients (T) and/or untreated patients (U). *: the comparisons using untreated patients as control were not included in the association’s analysis. Odds Ratio and its lower and upper limit are those from the included published systematic review.

| Author | IdP | Genotype | Antiepileptic | ADR | Control | Ethnicity | Odds Ratio | Lower limit | Upper limit |
| --- | --- | --- | --- | --- | --- | --- | --- | --- | --- |
| Chouchi (15) | 1 | HLA-B*15:02 | Carbamazepine | SCRs | T | Mostly Asian | 27.325 | 9.933 | 75.166 |
| Chouchi (15) | 2 | HLA-B*15:02 | Carbamazepine | SJS | T | Mostly Asian | 152.089 | 34.737 | 665.901 |
| Chouchi (15) | 3 | HLA-B*15:02 | Carbamazepine | SJS, TEN | T | Mostly Asian | 13.993 | 7.291 | 26.856 |
| Deng (33) | 4 | HLA-B*15:02 | Lamotrigine | SJS, TEN | T | Asian | 2.53 | 1.25 | 5.13 |
| Deng (33) | 5 | HLA-B*15:02 | Lamotrigine | MPE | T | Asian | 1.07 | 0.59 | 1.95 |
| Deng (33) | 6 | HLA-B*24:02 | Lamotrigine | SJS, TEN | T | Asian | 3.5 | 1.61 | 7.59 |
| Deng (33) | 7 | HLA-B*24:02 | Lamotrigine | MPE | T | Asian | 2.14 | 1.1 | 4.16 |
| Deng (33) | 8 | HLA-B*33:03 | Lamotrigine | MPE | T | Asian | 0.2 | 0.06 | 0.64 |
| Deng (33) | 9 | HLA-B*58:01 | Lamotrigine | MPE | T | Asian | 1.03 | 0.39 | 2.72 |
| Grover (16) | 10 | HLA-B*15:02 | Carbamazepine | SJS, TEN | T | Asian | 80.7 | 45.62 | 142.77 |
| Grover (16) | 11 | HLA-B*15:02 | Carbamazepine | HSS, MPE | T | Asian | 1.84 | 0.96 | 3.53 |
| Grover (16) | 12 | HLA-A*31:01 | Carbamazepine | SJS, TEN | T | Asian, European and American | 5.65 | 2.7 | 11.78 |
| Grover (16) | 13 | HLA-A*31:01 | Carbamazepine | HSS, MPE | T | Asian, European and American | 8.58 | 5.55 | 13.28 |
| Grover (16) | 14 | HLA-B*15:02 | Carbamazepine | SJS, TEN | U | Asian | 45.37 | 20.97 | 98.15 |
| Grover (16) | 15 | HLA-B*15:02 | Carbamazepine | HSS, MPE | U | Asian | 1.5 | 0.67 | 3.36 |
| Grover (16) | 16 | HLA-B*15:02 | Phenytoin | SJS, TEN | T | Asian | 5.26 | 2.51 | 11.04 |
| Grover (16) | 17 | HLA-B*15:02 | Lamotrigine | SJS, TEN | T | Asian | 3.59 | 1.15 | 11.2 |
| Grover (16) | 18 | HLA-B*15:02 | Valproate | SJS, TEN | T | Asian | 1.38 | 0.1 | 19.64 |
| Grover (16) | 19 | HLA-B*15:02 | Phenobarbital | SJS, TEN | T | Asian | 0.68 | 0.02 | 19.34 |
| Grover (16) | 20 | HLA-B*15:02 | Levetiracetam | SJS, TEN | T | Asian | 1 | 0.02 | 40.28 |
| Li (18) | 21 | HLA-B*15:02 | Phenytoin | SJS, TEN | T | Asian | 5.65 | 2.76 | 11.57 |
| Li (18) | 22 | HLA-B*15:02 | Lamotrigine | SJS, TEN | T | Asian | 4.51 | 1.57 | 12.98 |
| Tangamornsuksan (34) | 23 | HLA-B*15:02 | Carbamazepine | SJS, TEN | U&T | Asian | 79.84 | 28.45 | 224.06 |
| Wang (35) | 24 | HLA-B*40:01 | Carbamazepine | SJS, TEN | T | Asian | 0.22 | 0.14 | 0.35 |
| Wang (35) | 25 | HLA-B*15:11 | Carbamazepine | SJS, TEN | U | Asian | 11.11 | 2.62 | 47.09 |
| Wang (35) | 26 | HLA-B*15:11 | Carbamazepine | SJS, TEN | T | Asian | 17.43 | 3.12 | 97.41 |
| Wang (35) | 27 | HLA-B*46:01 | Carbamazepine | SJS, TEN | T | Asian | 0.49 | 0.25 | 0.95 |
| Wang (35) | 28 | HLA-B*58:01 | Carbamazepine | SJS, TEN | T | Asian | 0.23 | 0.09 | 0.58 |
| Wu (36) | 29 | CYP2C9*3 | Phenytoin | SJS, TEN | T | Asian | 8.93 | 2.63 | 30.36 |
| Wu (36) | 30 | CYP2C9*3 | Phenytoin | SJS, TEN | U | Asian | 8.86 | 5.23 | 15 |
| Yip (37) | 31 | HLA-B*15:02 | Carbamazepine | SJS, TEN | T | Asian | 113.39 | 51.24 | 250.97 |
| Yip (37) | 32 | HLA-A*31:01 | Carbamazepine | Hypersensitivity | T | Asian and European | 9.45 | 6.41 | 13.93 |
| Zeng et al (17) | 33 | HLA-B*15:02 | Lamotrigine | SJS, TEN | T | Asian | 4.98 | 1.43 | 17.28 |
