# Supplementary material for: Publication bias in pharmacogenetics of adverse reaction to antiseizure drugs: An umbrella review and a meta-epidemiological study
Source: PLoS One. 2022 Dec 30;17(12):e0278839. doi: 10.1371/journal.pone.0278839 (PMC9803138; doi:10.1371/journal.pone.0278839)
Supplement: Attachment — Submitted filename: PONE-D-21-20495 rev1_vf.docx [file pone.0278839.s003.docx]

PLOS ONE Decision: Revision required [PONE-D-21-20495] - [EMID:04cdbb83f48c962a]

Response to Reviewers

Dear Editor, Dear Reviewers,

Thank you very much for your time and advices. Please find below our corrections following your comments.

Of note, we realized that the initial calculation of the ratio of ORs, the ROR, omitted to take into account the variance of the adjusted ORs (OR_TM_). We corrected the corresponding results section “Exploration of the impact of publication bias” by removing the sentence “~~The interaction test for the association of [HLA-B*15:02-carbamazepine-SJS/TEN] (identification letter #C in Table 3) almost reach significativity (OR~~_~~NP~~_~~=39.77 [20.96; 75.48]; OR~~_~~TM~~_ ~~=21.15 [10.49; 42.67]; interaction test: ROR=1.88 [0.99; 3.57]).~~” and the table 3. Fortunately, this does not change the results, as the confidence intervals of the ROR were already not significant, they just became larger. We sincerely apologize for this mistake.

Best regards,

The corresponding author on behalf of the authors

Journal Requirements:

When submitting your revision, we need you to address these additional requirements.

2. We note that the grant information you provided in the ‘Funding Information’ and ‘Financial Disclosure’ sections do not match.

When you resubmit, please ensure that you provide the correct grant numbers for the awards you received for your study in the ‘Funding Information’ section.

-----

ANSWER

My bad, SB has not been funded by a specific project grant, it was a reference to its legally required traineeship grant. We corrected “The author(s) received no financial support for the research, authorship, and/or publication of this article.”

-----

3. Thank you for stating the following financial disclosure: "Funding
SB was funded by the UMR 5558, Université Lyon 1, CNRS (<https://www.univ-lyon1.fr/>).
The funders had no role in study design, data collection and analysis, decision to publish, or preparation of the manuscript."

-----

ANSWER

My bad, there was no specific funding. We corrected “The author(s) received no financial support for the research, authorship, and/or publication of this article.”

-----

We note that one or more of the authors is affiliated with the funding organization, indicating the funder may have had some role in the design, data collection, analysis or preparation of your manuscript for publication; in other words, the funder played an indirect role through the participation of the co-authors. If the funding organization did not play a role in the study design, data collection and analysis, decision to publish, or preparation of the manuscript and only provided financial support in the form of authors' salaries and/or research materials, please do the following:

a. Review your statements relating to the author contributions, and ensure you have specifically and accurately indicated the role(s) that these authors had in your study. These amendments should be made in the online form.

b. Confirm in your cover letter that you agree with the following statement, and we will change the online submission form on your behalf: 
“The funder provided support in the form of salaries for authors [insert relevant initials], but did not have any additional role in the study design, data collection and analysis, decision to publish, or preparation of the manuscript. The specific roles of these authors are articulated in the ‘author contributions’ section.

-----

ANSWER

My bad, there was no specific funding. We corrected “The author(s) received no financial support for the research, authorship, and/or publication of this article.”

-----

4. Thank you for stating the following in the Competing Interests section: "Conflict of interest 
SB, JC, MGC, GG declare that they have no competing interest. 
JCL has received speaking fees and honoraria from Roche.
CV has received consulting fees from Genzyme, Novartis and speaker honoraria from Galapagos.
SR received speaker and/or consulting fees from UCB Pharma, EISAI, GW Pharma, Idorsia, LivaNova, and Arvelle Therapeutics.
GL has received speaker honoraria from GWpharma, Eisai and Biomarin. 
MC has received consulting fees from Boehringer Ingelheim, SANOFI, AstraZeneca, EISAI and speaker honoraria from SANOFI."

We note that you received funding from a commercial sources: Roche, Genzyme, Novartis, UCB Pharma, EISAI, GW Pharma, Idorsia, LivaNova, Arvelle Therapeutics, Biomarin, Boehringer Ingelheim, SANOFI, and AstraZeneca.

Please provide an amended Competing Interests Statement that explicitly states this commercial funder, along with any other relevant declarations relating to employment, consultancy, patents, products in development, marketed products, etc.

-----

ANSWER

We added the PLOS ONE sentence “I have read the journal's policy and the authors of this manuscript have the following competing interests:”

-----

Within this Competing Interests Statement, please confirm that this does not alter your adherence to all PLOS ONE policies on sharing data and materials by including the following statement: "This does not alter our adherence to PLOS ONE policies on sharing data and materials.” (as detailed online in our guide for authors <http://journals.plos.org/plosone/s/competing-interests>).  If there are restrictions on sharing of data and/or materials, please state these. Please note that we cannot proceed with consideration of your article until this information has been declared.

Please include your amended Competing Interests Statement within your cover letter. We will change the online submission form on your behalf.

-----

ANSWER

We added the sentence “This does not alter our adherence to PLOS ONE policies on sharing data and materials.”

-----

5. In your Data Availability statement, you have not specified where the minimal data set underlying the results described in your manuscript can be found.

-----

ANSWER

The sentence “All relevant data are within the manuscript and its Supporting Information files.” Might be corrected as the following: “All relevant data are within the manuscript and its Supporting Information 4: Results of individual studies”

-----

6. Please include captions for your Supporting Information files at the end of your manuscript, and update any in-text citations to match accordingly. Please see our Supporting Information guidelines for more information: <http://journals.plos.org/plosone/s/supporting-information>.

-----

ANSWER

Done

-----

Reviewers' comments:

5. Review Comments to the Author

Reviewer #1: In the manuscript "Publication bias in pharmacogenetics of adverse reaction to antiepileptic drugs: an umbrella review and a meta-epidemiological study", the authors describe an umbrella review of systematic reviews and meta-analyses of pharmacogenetic associations with adverse drug reactions to antiepileptic medications. The authors limited their review to previously published meta-analyses and systematic reviews to identify gene-drug-adverse reaction associations with multiple studies. The authors were able to identify 22 distinct associations which were analyzed for publication bias using funnel plots and tests of asymmetry. Overall, the authors make an attempt at addressing a potentially important problem when making clinical guidelines with regard to pharmacogenetic risks when taking antiepileptics. I agree with the final conclusion that studies of pharmacogenetic interactions should likely be registered even if negative in order to provide complete data. There are several concerns from this reviewer, however.

1. There are multiple grammar and word choice errors throughout the manuscript that are distracting at best but may actually affect the author's meaning or intent in certain instances at worst. A thorough, careful editing is necessary to make the manuscript more readable and ensure clear communication to the reader.
-----

ANSWER

Thanks, we sent the paper to our university team for language editing.

-----
2. With regard to the author's methods, it is understandable that an umbrella review of meta-analyses and systematic reviews was utilized in order to exclude one-off pharmacogenetic interactions, but it is unclear why the authors did not conduct additional searches for specific interactions identified from the reviews in order to ensure that all publications were identified. Furthermore, if the intent was to exclude interactions with low numbers of publications, why were interactions with only one publication mentioned in a review included as identified interactions?
-----

ANSWER

Thanks, we added:

“We did not conduct additional searches of original clinical studies. Indeed, focusing on published systematic review allow assessing the impact of the publication bias in the currently available meta-analyses, which are used for guidelines elaboration. ”

And please see answer to question 3

-----
3. The authors state that 14 of 22 funnel plots were "not determinable" (line 231). Many of these "not determinable" funnel plots have low number of studies (table 2 and supplementary data). It was noted that all interactions with 1-4 studies were ND; 4 or less is studies is low enough to likely make funnel plot examination of little utility. It is unclear why these interactions were not simply outright excluded.
-----

ANSWER

Thanks, we added:

“We also did not remove the associations with a low number of estimates, leading to report results of little utility. Indeed, we did not consider exclusion criteria based on the number of point estimates. Therefore, we reported all the associations. Such exclusion criteria would require an arbitrary cut-off, which might be questionable. Above all, such exclusion criteria might lead to overestimate the prevalence of publication bias in the field. ”

-----
4. The reviewer is also unclear why interactions are studied so specifically. Given that there are cutaneous reactions of varying severity associated with overlapping genotypes and medications, would attempting a meta-analysis of all reactions for one variable (genotype, SJS) or combination of variable (e.g. SJS and HLA type) give better power to study publication bias in the field. On a related note, do the adverse drug reactions represent varying degrees of severity on a spectrum of cutaneous reactions or are the less severe reactions mediated by different mechanism and therefore have a specific dermatologic/histologic diagnosis? If they are all a similar mechanism, this would certainly allow for some logical grouping (e.g. all reactions in individuals with HLA-B*15:02 taking carbamazepine).
-----

ANSWER

Thanks, we added:

“Indeed, some logical grouping are probably legit and relevant. This would have increase the power of the analysis. For example, combining the association A, B and C ([HLA-B*15:02 – carbamazepine – SCR/SJS/SJS,TEN] allowed to reach a nominal p value <0.05 for both the tests of Egger (p = 0.0009) and of Begg (p = 0.02) (supplementary figure S2). However, this would have probably lead to overestimate the prevalence of the publication bias in the field. Therefore,… ”

-----
5. It is my understanding regarding the Egger's test that it is appropriate for randomized controlled trials but it is unclear if it is appropriate for studying pharmacogenetic interactions. The authors briefly mention in limitations that there are no established methods for studying publication bias in pharmacogenetics but this point is not specifically addressed.

-----

ANSWER

Thanks, we added:

“Moreover, tools for publications bias assessment, as the Egger's test for example, have been initially developed for meta-analysis of randomized trials. However, most of the available pharmacogenetic studies here were non-randomized. If the same tools for publications bias assessment may be used in meta-analysis of such non-randomized studies remains unclear”

-----

Reviewer #2: This is very interesting paper addressing very important issue on publication bias in pharmacogenetic biomarkers (PGx) and antiepileptics adverse drug reactions (ADRs). This is very important topic and yet, have not received very much attention. The authors have confirmed that publication bias in the pharmacogenetic of antiepileptic ADRs is not uncommon and may affect the estimation of the effect of such biomarkers. I have a minor question that needs a clarification.
The results from this present study suggested that there is a significant publication bias for the HLA-B*15-2 and its association with the risk of carbamazepine-induced SCARs. Could you please clarify how to interpret this bias despite the fact that the use of HLA-B*15-02 screening has led to dramatically decrease in the incidence of SJS/TEN in Southeast Asia? How should we use this data in clinical practice?

-----

ANSWER

Thanks, we added:

“The effect size of these associations remain high (from ≈4 to ≈40), not negating their use in clinical practice. ”

-----
