# Supplementary material for: Publication bias in pharmacogenetics of adverse reaction to antiseizure drugs: An umbrella review and a meta-epidemiological study
Source: PLoS One. 2022 Dec 30;17(12):e0278839. doi: 10.1371/journal.pone.0278839 (PMC9803138; doi:10.1371/journal.pone.0278839)
Supplement: Attachment — Submitted filename: Response to Reviewers.docx [file pone.0278839.s004.docx]

PONE-D-21-20495R1_rev2

Dear Editor, Dear Reviewers,

Thank you very much for your time and advices. Please find below our corrections following your comments.

Best regards,

The corresponding author on behalf of the authors

6. Review Comments to the Author

Reviewer #1: (No Response)

-----

ANSWER: NA

-----

Reviewer #3: In the manuscript entitled "Publication bias in pharmacogenetics of adverse reaction to antiseizure drugs: an umbrella review and a meta-epidemiological study", the author analysed the error estimation of the correlation between pharmacogenetic biomarkers and antiseizure drug's adverse effects based on the discussion of publication bias. This is a very interesting article, and the research idea is very creative. After a revision of this manuscript, there is no obvious loophole in the article. Author's conclusions drawn seemed to be unbiased with rigorous methodology, therefore, I just give some minor comments on it.

-----

ANSWER: thank you very much!

-----

1) The author only selected Medline database in the retrieval process, but did not seem to explain the specific reasons. I am afraid that some literature may be missing. Although the author uses other methods to supplement the search results except database retrieval, I believe that multiple relevant databases should be used in the retrieval process to avoid omission.

-----

ANSWER: Indeed, we added the following in the methods section:

“We limited the information source to the Medline database. Indeed, the Clinical Pharmacogenetics Implementation Consortium (CPIC) Guideline for HLA genotype and Use of Carbamazepine and Oxcarbazepine was restricted to the use of “the PubMed database”^11^. Using the same restriction allowed to estimate the impact of publication bias in the context of the current practice of guidelines elaboration in this area.”

-----

2) The Egger and Begg's methods are the most commonly used methods for funnel chart symmetry test, but publication bias is not the only source of asymmetry, such as methodological design and statistical analysis methods may affect it. Although the author mentioned this in the chapter of research limitations, I want to know the author's views on this issue, because in my opinion, this issue can reduce its impact on the results through some methods.
This question just makes me curious, because I have consulted some methodological articles, and the relevant items mentioned in them are not applicable to the author's research methods. I just made some guesses about whether subgroup analysis or meta regression can solve this problem.

-----

ANSWER: Indeed, we added the following in the discussion section:

“Moreover, publication bias is not the only source of funnel plot asymmetry. Especially, a poor methodological quality of the small-included studies might led to the “small study effect”. A true heterogeneity also might contribute to the funnel asymmetry. However, in our study, some of the punctual estimates seem to line up with usual cut-off p values for significance (see notably figure 2, along the 1% cut off); the use of contour-enhanced funnel plots allowed to highlight that the asymmetry seems to be associated with the significance of the included studies. Moreover, we believed that the heterogeneity is limited in our funnel plots, as we kept the most precise granularity, notably by not gathering different, despite close, adverse drug reactions, in line with the author’s definitions.”

-----
